# Case literature analysis of Fournier’s gangrene caused by sodium-glucose protein-2 inhibitors

**DOI:** 10.3389/fmed.2024.1301105

**Published:** 2024-04-12

**Authors:** Hailing Liu

**Affiliations:** Department of Pharmacy, Hunan Provincial People's Hospital, Changsha, China

**Keywords:** SGLT-2 inhibitor, Fournier’s gangrene, adverse drug reaction, literature analysis, case

## Abstract

**Objective:**

To analyze the clinical characteristics and correlation of Fournier’s gangrene induced by sodium-glucose cotransporter protein-2 (SGLT-2) inhibitors, providing references for safe clinical drug use.

**Methods:**

The CNKI, WanFang, and PubMed databases were searched, and relevant documents were collected and statistically analyzed. The basic information of patients, drug use information, adverse reactions and outcomes were extracted and analyzed.

**Results:**

A total of 12 patients (8 males and 4 females) were included, with an average age of 55.6 years (ranging from 34 to 72 years). SGLT-2 inhibitors associated with Fournier’s gangrene include empagliflozin (5 cases), dapagliflozin (5 cases), and canagliflozin (2 cases). Among them, 10 cases reported the time of first medication, ranging from 1 month to 6 years for the occurrence of adverse reactions. The most common concomitant drug was metformin (7 cases). Adverse reactions mainly manifested as redness, swelling and pain in the buttocks, perineum, perianal, scrotum and other positions, accompanied by an increased white blood cell count. Following surgery and antibiotic treatment, all patients showed improved.

**Conclusion:**

Fournier’s gangrene induced by SGLT-2 inhibitors is rare. If patients using SGLT-2 inhibitors are suspected of having Fournier’s gangrene, it is recommended to discontinue the drugs immediately and initiate active treatment to ensure clinical safety.

## 1 Introduction

SGLT-2 are common hypoglycemic drugs in clinical practice and are among the cornerstone drugs in the treatment of heart failure. SGLT-2 inhibitors reduce glucose and sodium reabsorption by inhibiting SGLT-2 in the proximal convoluted tubular epithelial cell membrane, thereby excreting large amounts of glucose in the urine (1). However, an increased urinary glucose concentration can elevate the risk of urethral and genital infections (2).

Fournier’s gangrene (FG) is one of the rare but highly progressive adverse reactions. FG, also known as perineal necrotizing fasciitis, can penetrate soft tissue structures and destroy subcutaneous fat and muscle, leading to necrosis of perineal, perianal, and genitourinary areas, and the case fatality rate can be as high as 50% (3). The most common pathogenic cause of FG is infection of adjacent tissues, such as abscesses, anal fissure, and colonic perforation.

In 2018, the Food and Drug Administration (FDA) issued a safety warning on SGLT-2 inhibitors regarding FG in patients with type 2 diabetes. In January 2022, the American Diabetes Association identified 491 cases of FG associated with SGLT-2 inhibitors (3). Despite the issuance of a warning for the risk of FG with SGLT-2 inhibitors, the number of related cases continued to rise (3). Therefore, this study conducted a comprehensive analysis by searching relevant case reports for the reference of clinical users.

## 2 Materials and methods

### 2.1 Retrieval strategy

Literature search for Fournier’s gangrene caused by SGLT-2 inhibitors included databases such as CNKI, Wanfang, and PubMed. English search terms comprised “Fournier’s gangrene,” “SGLT-2,” “SGLT,” “empagliflozin,” “dapagliflozin,” “canagliflozin,” “ipragliflozin,” “ertugliflozin,” “luseogliflozin,” “ofogliflozin,” and “Sodium-Glucose Cotransporter-2 Inhibitors.” The search was completed as of February 12, 2023.

### 2.2 Inclusion and exclusion criteria

Inclusion criteria: We collected original case reports of FG caused by SGLT-2 inhibitors.

Exclusion criteria: Reviews, repeated cases, cases where the causal relationship between SGLT-2 inhibitors and FG cannot be determined, and cases lacking full texts were excluded.

### 2.3 Data collection

Read the included literature in detail and record the patient’s age, gender, Region, chief complaint, past history, white blood cell count (WBC), C-reactive protein, hemoglobin, glycosylated hemoglobin, glucose, bacterial culture, culture sample, treatment process, time of first medication and adverse reaction, outcome and other information.

## 3 Results

### 3.1 Identified studies

A total of 24 articles were retrieved, and 12 articles were screened based on the inclusion criteria and exclusion criteria, resulting in a total of 12 cases.

### 3.2 Basic information

A total of 12 patients were included, including 8 men and 4 women, with a median age of 55.6 years (range 34–72) (Table 1). Among them, 5 patients were treated with empagliflozin, 5 with dapagliflozin, and 2 with canagliflozin. Ten patients reported the time of first medication, with the shortest duration being 1 month and the longest 5 years, occurring more than 6 months after medication. Five patients reported the specific dosage of SGLT-2 inhibitors, including empagliflozin at dose of 10 mg, 12.5 mg, and 25 mg/d, dapagliflozin at 10 mg/d, and canagliflozin at 100 mg/d. Following treatment, all patients showed improvement.

**Table 1:** The basic information of the 12 included patients.

Study	Region	Time	Age	Gender	SGLT-2 inhibitors	Dosage	Adverse reactions	Outcome
Jahir et al. (4)	America	2022	58	Woman	Empagliflozin	–	–	WBC was reduced
Khokhar et al. (5)	America	2022	55	Man	Empagliflozin	12.5 mg/d	88d	Normal
Vargo et al. (6)	America	2021	64	Man	Dapagliflozin	–	1.5 years	Normal
Elbeddini et al. (7)	Canada	2020	72	Man	Canagliflozin	–	6 years	Normal
Garcia-Garcia et al. (8)	Spain	2020	68	Man	Dapagliflozin	10 mg/d	19 months	Normal
Kasbawala et al. (9)	America	2020	37	Woman	Canagliflozin	100 mg/d	1 month	Normal
Ellegard and Prytz (10)	Sweden	2020	52	Woman	Dapagliflozin	–	1.5 years	Normal
Elbeddini et al. (11)	Canada	2020	71	Woman	Dapagliflozin	–	5 years	Normal
Lindsay et al. (12)	America	2020	51	Man	Empagliflozin	25 mg/d	–	Normal
Nagano et al. (13)	Japan	2019	34	Man	Empagliflozin	10 mg/d	142 d	Normal
Onder et al. (14)	Turkey	2018	64	Man	Dapagliflozin	-	6 months	Normal
Kumar et al. (15)	Australia	2017	41	Man	Empagliflozin	25 mg/d	14 months	Normal

### 3.3 Clinical symptoms

The main manifestations of adverse reactions include redness and pain in the buttocks, perineum, perianal, scrotum, and other positions, accompanied by an increase in white blood cell count (Tables 2, 3). Pain affects sleep and may even lead to fever, requiring timely intervention and treatment.

**Table 2:** Chief complaint, complications, and drug combinations of the 12 included patients.

Study	Chief complaint	Complication	Drug combination
Jahir et al. (4)	Within a week, severe pain and swelling appeared in the upper right thigh and perineum	Type 2 diabetes, hypertension, diabetic Ketoacidosis and hyperlipidemia	–
Khokhar et al. (5)	Within a week, the ulcer in the right groin area appeared and worsened.	Type 2 diabetes, hypertension, and AIDS	Symtuza
Vargo et al. (6)	There was erythema, blisters and palpation in the left scrotum	Type 2 diabetes, atrial fibrillation, coronary heart disease	Metformin, warfarin, and aspirin
Elbeddini et al. (7)	Rectal pain, diarrhea	Type 2 diabetes, hemorrhoids, and prostate cancer	Metformin, sitagliptin, and insulin glargine
Garcia-Garcia et al. (8)	Syncope, head injury, and fever	Type 2 diabetes, hypertension, dyslipidemia, heart disease, prostate cancer	Metformin, sitagliptin, and insulin glargine
Kasbawala et al. (9)	Left buttock pain was associated with dysuria	Type 2 diabetes, diabetic Ketoacidosis	Metformin, sitagliptin
Ellegard and Prytz (10)	Fever for 3 days, and swelling in the breech region and pain worsened	Type 2 diabetes, obesity, hypertension, asthma, and hepatitis B	Insulin, and prednisolone
Elbeddini et al. (11)	Anal discomfort	Type 2 diabetes, hypertension, and hyperlipidemia	Glimepiride, linagliptin, Tundopril, amlodipine, bisoprolol, and rosuvastatin
Lindsay et al.(12)	Discomfort and perianal pain	Type 2 diabetes mellitus, hypertension, diabetic Ketoacidosis and hyperlipidemia	Metformin, lisinopril, atorvastatin, and aspirin
Nagano et al. (13)	Perineal and groin pain and swelling for 3 days	Type 2 diabetes	Sitagliptin, Glibenclamide
Onder et al. (14)	Scrotal pain, swelling, and redness	Type 2 diabetes, and painful hemorrhoids	Metformin, vildagliptin, and insulin
Kumar et al. (15)	Pain in scrotum	Type 2 diabetes mellitus, diverticulum perforation, and oral thrush	Metformin

**Table 3:** Treatments of the 12 included patients.

Study	WBC	Bacteria cultures	Culture samples	Treatments
Jahir et al. (4)	26.6*10^^3^/μL	Gram-positive cocci, Streptococcus green and Corynebacterium	Blood and urine, wounds	Broad-spectrum antibiotics
Khokhar et al. (5)	13*10^^9^/L	Streptococcus angina, *Staphylococcus epidermidis*	Wound	Empirical antibiotic therapy; later switched to ampicillin sulbactam
Vargo et al. (6)	–	–	–	Broad-spectrum antibiotics
Elbeddini et al. (7)	17.8*10^^9^/L	*Bacteroides ovatus*, Prevotella denti-cola and Actinomycetes	Wound	Empirical antibiotic therapy, followed by sulfamethoxazole, trimethoprim, ciprofloxacin, and metronidazole.
Garcia-Garcia et al. (8)	23,200/mm^3^	Extended-spectrum β-lactamase *E. coli* and *Pseudomonas aeruginosa*; *Escherichia coli*, *Pseudomonas aeruginosa*, *morganella morganii*, and enterococcus	urine	Empirical antibiotic therapy.
Kasbawala et al. (9)	–	–	–	Ceftazidime, clindamycin and vancomycin were started; then vancomycin and piperacillin/tazobactam
Ellegard and Prytz (10)	19*10 ^^9^/L	A combination of aerobic and anaerobic pathogens	Fat and muscle tissue	Broad-spectrum antibiotics; later changed to meropenem and clindamycin.
Elbeddini et al. (11)	33.2*10^^ 9^/L	Gram-positive cocci, Gram-negative bacilli, and Gram-positive bacilli	The perineal swab	Vancomycin, piperacillin-tazobactam and clindamycin; vancomycin was suspended later
Lindsay et al. (12)	20,000/μL	–	–	Broad-spectrum antibiotic
Nagano et al. (13)	21.7*10^^9^/L	Methicillin-resistant *Staphylococcus aureus* (MRSA)	Tissue	Change from meropenem and clindamycin to vancomycin
Onder et al. (14)	29.6*10^^3^/μL	–	–	Ceftriaxone and metronidazole
Kumar et al. (15)	18.3*10^^9^/L	Streptococcus angina, mixed anaerobes, and gram-negative bacilli, with a large number of multiple microorganisms	Operative site	Amoxicillin, gentamycin and vancomycin; later changed to meropenem

### 3.4 Complication and the combination of medication

All cases were diabetic patients. In addition, the most common complications were hypertension, hyperlipidemia, diabetic ketoacidosis, prostate cancer, and obesity, with 6, 4, 3, 2, 2 cases, respectively. Out of the 12 patients, 11 were taking concomitant medications, of which 7 were prescribed metformin (Table 2). When combining these diseases or using metformin in combination, it is important to monitor patients for any adverse reactions.

### 3.5 Laboratory test

Laboratory tests are summarized in Table 4. Nine cases reported elevated glycated hemoglobin levels, with eight exceeding the normal range. Seven cases reported blood glucose and six of them were higher than the normal range. When patients experience FG, blood sugar management is inadequate.

**Table 4:** Laboratory tests of the 12 included patients.

Study	Glycosylated Hemoglobin	Glucose
Jahir et al. (4)	–	12.7 mmol/L
Khokhar et al. (5)	8.20%	–
Vargo et al. (6)	–	–
Elbeddini et al. (7)	7.50%	–
Garcia-Garcia et al. (8)	7.80%	–
Kasbawala et al. (9)	9.80%	22.3 mmol/L
Ellegard and Prytz (10)	–	26.3 mmol/L
Elbeddini et al. (11)	11.70%	–
Lindsay et al. (12)	9.00%	17.5 mmol/L
Nagano et al. (13)	6.50%	6.1 mmol/L
Onder et al. (14)	7.40%	14.4 mmol/L
Kumar et al. (15)	11.20%	19.9 mmol/L

### 3.6 Treatments

Treatment involves discontinuation of the medication, urgent and aggressive surgical exploration with debridement of necrotic tissue, and administration of broad-spectrum antibiotics, among other measures (16). All patients underwent surgical debridement. White blood cell count was reported in 10 patients, all above normal values (9.5 × 10^9/L) (Table 3). Bacteria cultures were performed in eight patients. Culture samples involved blood samples, urine samples, wound sampling, perineal swabs, and surgical site sampling; a variety of bacterial species were cultured, including Gram-positive cocci and Gram-negative rods (Table 3). The patient was admitted immediately admitted on empirical antibiotic intravenous therapy including vancomycin, meropenem, clindamycin, ampicillin sulbactam, daptomycin, ceftazidime, piperacillin/tazobactam, ceftriaxone, metronidazole, amoxicillin, and gentamicin. Later, the medication was adjusted according to the patient’s condition and the results of bacterial culture, and the type of antibiotics or prescription were changed.

## 4 Discussion

FG, also known as perineal necrotizing fasciitis, is a urinary emergency characterized by progressive necrotic infection of the external genitalia or perineum, local skin purple-black or brown, exuding large amounts of purulent fluid, accompanied by a foul odor, subcutaneous spasm, rare but rapid onset. Diabetes is an important predisposing factor, along with obesity, cancer, advanced age, and other immunosuppressive diseases (17). Management relies on early identification. Treatment includes discontinuation of medication, urgent and aggressive surgical exploration with debridement of necrotic tissue, and administration of broad-spectrum antibiotics, among other measures (16).

SGLT-2 inhibitors, including canagliflozin, dapagliflozin, empagliflozin, and ertugliflozin, were first approved in 2013 for adults with type 2 diabetes. They exert a hypoglycemic effect by promoting the excretion of sugar through the kidneys. Both the UK Medicines and Healthcare Products Regulatory Agency (MHRA) (18) and the US FDA (19) have concluded that the risk of FG for SGLT-2 inhibitors is warranted based on received case reports. The cases included in this study were reported from 2017 to 2022, totaling 12 cases. Bershoff-Matcha et al. identified a total of 55 cases meeting the inclusion criteria in the FAERS database, with a median age of 56 years, 39 males, and 16 females. The average time to event was 9 months (range: 5 days to 49 months). Reported complications included diabetic ketoacidosis, and all patients underwent surgical debridement (20). As of January 2022, the American Diabetes Association identified a total of 491 cases of FG associated with SGLT-2 inhibitors, of which 162 were caused by canagliflozin, 101 cases of dapagliflozin, and 223 cases of empagliflozin and found a statistically significant increase in the risk of FG hospitalization with SGLT2i treatment compared to the use of two or more non-SGLT2 inhibitors or insulin therapy alone (3). Bao Anh Tran et al. reported a median age of 54 years and 52 years in cases from the FAERS database and literature review, respectively. In both datasets, the incidence of FG was higher in males than females. Other reported data included clinical outcomes and concomitant antihyperglycemic medications, which are consistent with the findings of my study. In addition, the number of associated cases continues to rise despite the issuance of a FG risk warning for SGLT-2 inhibitors (3). Therefore, both physicians and patients should be concerned about the adverse effects of SGLT-2 inhibitors. SGLT-2 inhibitors’ mechanism in causing Fournier’s gangrene (FG) is still unclear, potentially associated with the stimulation of urinary glucose excretion (1), SGLT-2 inhibitors function by inhibiting the renal tubular reabsorption of glucose, leading to a significant excretion of glucose in the urine. This high concentration of urinary glucose can create an ideal environment for bacterial growth, thereby increasing the risk of gangrene (2). Additionally, the presence of urinary glucose may lead to localized osmotic changes, contributing to bacterial infection (21). On the other hand, SGLT-2 inhibitors might exert some influence on the immune system, but the specific mechanism remains unclear (22). Of the 12 patients included in this review, all had diabetes, and 11 of them exhibited poor glycemic control at the onset of FG (glycosylated hemoglobin, HbAlc >7.0%), so there was still a risk of FG despite good glycemic management with SGLT-2 inhibitors.

According to the guidelines for the clinical application of antimicrobial drugs issued in 2015, the selection of antibacterial drug varieties should in principle be determined according to the results of bacterial drug susceptibility tests, and the corresponding qualified specimens should be retained in time before starting antimicrobial treatment for pathogenic testing. Among the cases included in this paper, 8 patients underwent bacterial culture, and the culture rate accounted for only 66.7%, suggesting that the awareness of bacterial drug susceptibility testing among medical workers should be strengthened. In this study, cultures showed that patients with FG were monocultures or a combination of bacteria of aerobic, anaerobic, or facultative anaerobic bacteria. In mixed infection, aerobic bacteria can provide a suitable growth environment for anaerobic bacteria after consuming oxygen, while producing leukocyte toxins to avoid phagocytosis of white blood cells, and its metabolites can also lead to endarteritis obliterans, making the skin and subcutaneous tissue ischemic necrosis, resulting in the further spread of pathogenic microorganisms.

This study identified 12 case reports from literature retrieval, which is more than found in any other articles. Furthermore, the study results support previous findings, providing a comprehensive summary of clinical characteristics, and conducting a thorough analysis and discussion. However, due to the nature of being case reports, the level of evidence is relatively low. Additionally, it is challenging to determine whether the occurrence of FG in patients using SGLT2 inhibitors is a direct correlation. Therefore, careful consideration of potential risk factors for FG in patients is warranted.

## 5 Conclusion

In summary, FG caused by SGLT-2 inhibitors, despite its low incidence, is rapidly progressive and severe. When SGLT-2 inhibitors are used clinically, the patient’s discomfort during medication should be closely observed, and if FG is suspected, it is recommended to stop the drug immediately and start broad-spectrum antibacterial drugs and surgical debridement combination therapy to ensure clinical safety. For patients progressing to diabetic ketoacidosis, the insulin injections and aggressive fluid resuscitation are also necessary.

## Data availability statement

The original contributions presented in the study are included in the article/supplementary material, further inquiries can be directed to the corresponding author.

## Ethics statement

Ethical approval was not required for the study involving humans in accordance with the local legislation and institutional requirements. Written informed consent to participate in this study was not required from the participants or the participants’ legal guardians/next of kin in accordance with the national legislation and the institutional requirements.

## Author contributions

HL: Writing – original draft, Writing – review & editing.
